# TGF-β1 Reduces miR-29a Expression to Promote Tumorigenicity and Metastasis of Cholangiocarcinoma by Targeting HDAC4

**DOI:** 10.1371/journal.pone.0136703

**Published:** 2015-10-06

**Authors:** Huiling Wang, Caixia Li, Zhixiang Jian, Yingliang Ou, Jinrui Ou

**Affiliations:** 1 General Surgery Department of Guangdong General Hospital & Guangdong Academy of Medical Sciences, Guangzhou, Guangdong 510080, China; 2 Department of Rheumatology, The First Affiliated Hospital/School of Clinical Medicine of Guangdong Pharmaceutical University, Guangzhou, Guangdong 510080, China; H.Lee Moffitt Cancer Center & Research Institute, UNITED STATES

## Abstract

Transforming growth factor β1 (TGF-β1) and miRNAs play important roles in cholangiocarcinoma progression. In this study, miR-29a level was found significantly decreased in both cholangiocarcinoma tissues and tumor cell lines. TGF-β1 reduced miR-29a expression in tumor cell lines. Furthermore, anti-miR-29a reduced the proliferation and metastasis capacity of cholangiocarcinoma cell lines in vitro, overexpression of miR-29a counteracted TGF-β1-mediated cell growth and metastasis. Subsequent investigation identified HDAC4 is a direct target of miR-29a. In addition, restoration of HDAC4 attenuated miR-29a-mediated inhibition of cell proliferation and metastasis. Conclusions: TGF-β1/miR-29a/HDAC4 pathway contributes to the pathogenesis of cholangiocarcinoma and our data provide new therapeutic targets for cholangiocarcinoma.

## Introduction

Cholangiocarcinoma, arising from the bile duct epithelium, is a malignant tumor associated with high mortality rates. The incidence of cholangiocarcinoma is increasing worldwide[1]. Because of late diagnosis and early metastasis, surgical resection and conventional chemotherapy do not effectively prolong long-term survival[2]. It is thus necessary to identify a new means of early diagnosis and effective treatment of cholangiocarcinoma.

The transforming growth factor (TGF)beta family, which consists of three isoforms within mammary tissues(TGF-β1, TGF-β2 and TGF-β3), regulates tumor initiation, progression and metastasis[3,4]. TGF-β1 expression is more abundant than the other two isoforms during tumorigenesis[5]. Increasing evidence demonstrates that dysregulation of TGF-β1 has been identified in several cancers[6–10], including cholangiocarcinoma[6,7]. However, the biological effects for TGF-β1 in the development of cholangiocarcinoma has not been fully elucidated.

Besides TGF-β signaling, many other cytokines and signaling pathways are also associated with the modulation of cancer progression and metastasis. MicroRNAs (miRNAs), 18 to 25 nucleotides in length, are noncoding RNAs mediating degradation of specific mRNA targets via sequence-specific interactions with the mRNA 3′ untranslated regions (UTRs) [11]. MiRNAs play critical roles in many physiological and pathological processes, including proliferation, apoptosis, invasion and migration[12–16]. Aberrant miRNAs expression are involved in cholangiocarcinoma development, such as miR–21, miR–370, miR–373 and miR–200[17–21].

miR-29a has emerged as a potential tumor suppressor in multiple human neoplasms. Several studies have shown that miR-29a was significantly downregulated in gastric, lung, and hepatocellular cancer[22–24]. In gastric cancer, it can inhibit cell proliferation and induce cell cycle arrest by downregulating p42.3. Exogenous overexpression of miR-29a can significantly reduce cell proliferation and tumor formation in vivo in lung cancer. Consistent with these observations, we found decreased expression of miR-29a in cholangiocarcinoma tissues compared with matched non-neoplastic tissues. However, the molecular mechanism responsible for the dysregulation of miR-29a in cholangiocarcinoma remains largely unknown.

Growing evidence supports the interactions between TGF-β1 and miRNAs, however, whether and how TGF-β1 could regulate miR-29a during cholangiocarcinogenesis process remains to be determined. Here, we demonstrated that miR-29a was significantly reduced in cholangiocarcinoma cells and tissues. Furthermore, miR-29a inhibited cholangiocarcinoma cell growth and metastasis by targeting HDAC4. Our findings will help to elucidate the roles of TGF-β1/miR-29a/HDAC4 in the pathogenesis of cholangiocarcinoma and provide new therapeutic targets.

## Materials and Methods

### Cell lines and tissue samples

The human cholangiocarcinoma cell line FRH–0201, CCLP–1 and the human intrahepatic bile duct epithelial cell line HIBEC, which were all purchased from American Type Culture Collection(ATCC, USA), were cultured in RPMI–1640 (Gibco, USA) supplemented with 10% (v/v) fetal bovine serum (Gibco, USA) at 37°C in a humidified chamber under 5% (v/v) CO2.

Forty matched samples of cancer-adjacent tissue and cholangiocarcinoma tissue were obtained from patients undergoing surgical resection at the Guangdong General Hospital, after written informed consent was obtained from all patients. The matched cancer- adjacent samples were obtained at least 5 cm away from the tumor site. This study was approved by the Research Ethics Committee of Guangdong General Hospital (The approval number was GDREC2015097H).

### Quantitative real-time RT-PCR (qRT-PCR) analysis

Total RNA was extracted from cells using the TRIzol reagent (Invitrogen, USA) and cDNA was synthesized using a cDNA synthesis kit (TaKaRa, Japan) with 1μg amounts of total RNA as templates. qRT-PCR was performed with the aid of an SYBR Green PCR kit (TaKaRa, Japan) and an Applied Biosystems 7500 Sequence Detection System, following the manufacturers’ protocols. β-actin served as the internal control. The specific primers used to amplify HDAC4 were: Forward: 5′- CGCACAGTCCTTGGTTGG–3′ and reverse: 5′-CTGCTGGAACTGCTGCTTG–3′; the β-actin specific primers were 5′-ACTCGTCATACTCCTGCT–3′ (forward) and 5′-GAAACTACCTTCAACTCC–3′ (reverse).

MicroRNA quantitative RT-PCR was performed using an All-in-One First-Strand cDNA Synthesis Kit (GeneCopoeia) and a Hairpin-it miRNA qPCR Quantitation Kit (GenePharma); U6 served as the miRNA control. Each sample was measured in triplicate, and gene expression levels were calculated using the 2^-△△Ct^ method.

### Cell transfection

Cells were transfected using the X-tremeGENE siRNA Transfection Reagent (Roche, China) following the protocol of the manufacturer. Cells were seeded into six-well plates at 30% confluence on the day prior to transfection. MiR-29a mimic (sense, 5’-UAGCACCAUCUGAAAUCGGUUA–3’; antisense, 5’-ACCGAUUUCAGAUGGUGCUAUU–3’) and inhibitor (5’-UAACCGAUUUCAGAUGGUGCUA) were obtained from GenePharma (shanghai,China). To regulate miR-29a expression, both mimics (50 nM) and inhibitors (100 nM) of miR-29a were transfected. Overexpression of HDAC4 was performed by transfecting pcDNA3.1-HDAC4 as described previously[25].

### Cell proliferation assay

Cells were plated in 96-well plates at 5,000 /well, after 24 h of incubation, next transfected with miR-29a, an miR control, or siHDAC4 respectively. Cells were cultured for a further 24, 48, or 72 h, and then analyzed using a Cell Counting Kit–8 (Dojindo, Japan), according to the manufacturer’s instructions.

### Cell migration and invasion

To investigate the migration and invasion of cholangiocarcinoma cells in vitro, wound healing assay and transwell assay were performed respectively.

For wound healing assay, tumor cells in 12-well plates (11,000 cells per well) were transfected with miR-29a mimic or NC, scratch wounds were made using a sterilized 100ul plastic pipette tip. The width of wounds were measured under inverted microscope and wound- healing percentage was calculated.

For transwell assay, the membrane was coated with Matrigel in advance.1640 supplemented with 10%FBS was added to the lower chamber. Tumor cells were resuspended in serum-free 1640 (5×10^5^cells/ml) and 200μL was added to the upper chambers. After 8 hours incubation, cells which migrated to the lower face of the membrane were fixed with methanol and stained by crystal violet. After being washed by PBS for 3 times, the invasion rate was measured by counting the migrating cells under the microscope.

### DNA constructs and the dual-luciferase reporter assay

The pmirGLO Dual-Luciferase miRNA target expression vector, which uses firefly luciferase (luc2) as the primary reporter and *Renilla* luciferase (hRluc-neo) as the control reporter, was purchased from Promega. To construct plasmid pmirGLO-wt-HDAC4 (containing the wild-type HDAC4 3′-UTR binding site), 259 bp of the HDAC4 3′-UTR were PCR-amplified from human genomic DNA and cloned into the pmirGLO vector between the *Sac*I and *Sal*I restriction sites. To construct plasmid pmirGLO-mHDAC4 (containing a mutant HDAC4 3′-UTR), a mutation identified using Targetscan (http://www.TargetScan.org/) was induced using a Site-Directed Mutagenesis kit (SBSGenetech, China), following the manufacturer’s instructions. One day after transfection, luciferase activity levels were measured using the dual-luciferase reporter assay system (Promega) according to the manufacturer’s instructions. Each transfection was performed in triplicate.

### Western blotting

Different groups of cells were lysed in RIPA lysis buffer (keyGEN), and total protein concentration was measured by BCA assay kit (KeyGEN). Western blotting was performed as described previously [24] with rabbit anti-TGFβ1 pAb (dilution 1:500, BOSTER), rabbit anti-HDAC4 mAb (dilution 1:1,000, CST), rabbit anti-vimentin mAb (dilution 1:1,000, CST), rabbit anti- E-Cadherin mAb (dilution 1:1,000, CST), rabbit anti-MMP–2 mAb (dilution 1:1,000, CST) and rabbit anti-MMP–9 mAb (dilution 1:1,000, CST), whereas a rabbit anti-β-actin monoclonal antibody (dilution 1:1,000, CST) detected the internal control protein.

### Statistical analysis

Statistically significant differences between the two groups were identified using Student’s *t* test. Data are expressed as means ± standard deviation (SD) of at least three independent experiments. P<0.05 was considered to reflect statistical significance.

## Results

### Expression of miR-29a was reduced in human clinical cholangiocarcinoma samples and cell lines

**Fig 1:**
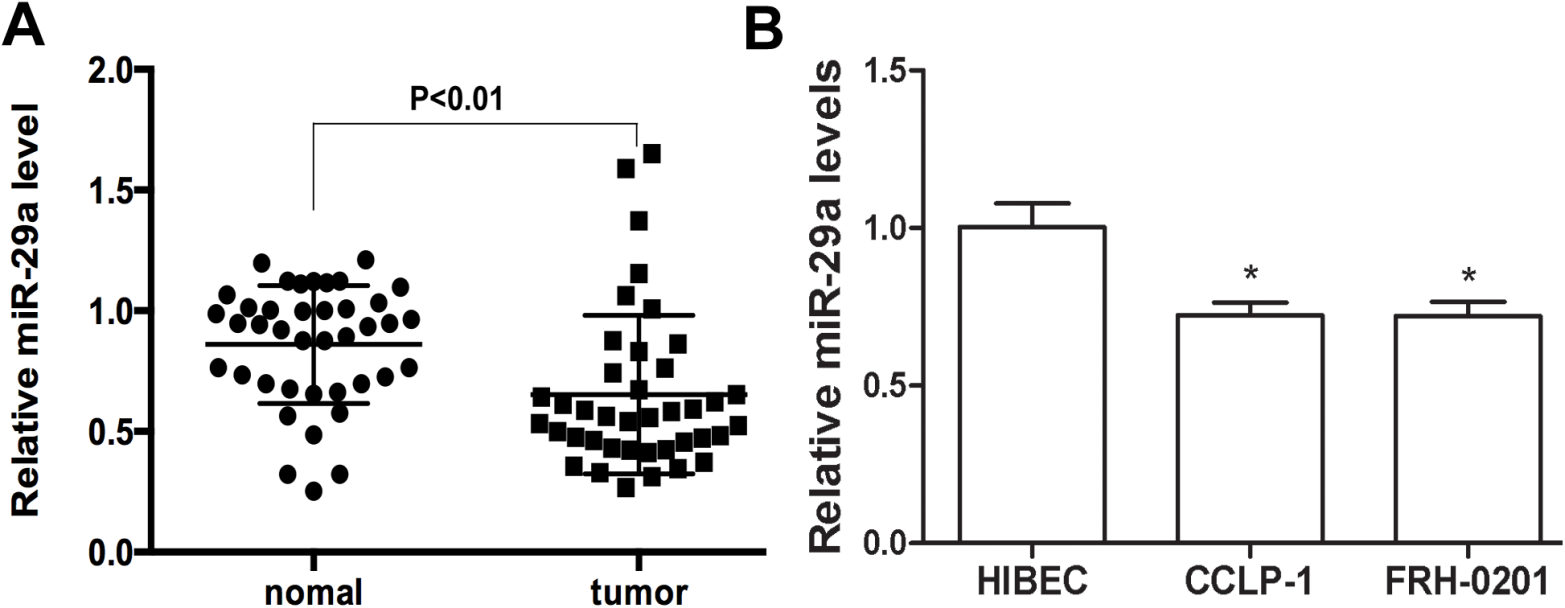
The expression of miR-29a was decreased in cholangiocarcinoma samples and cell lines. (A) The average expression level of miR-29a was measured in forty human cholangiocarcinoma tissues and matched cancer-adjacent (normal) tissues. (B) Expression of miR-29a in the human intrahepatic bile duct epithelial cell line HIBEC and two cholangiocarcinoma cell lines. Data are shown as mean±SD; *P<0.05.

The expression of miR–29 family was first examined by quantitative real-time PCR(qRT-PCR) in 40 matched cholangiocarcinoma and cancer-adjacent tissues. As shown in Fig 1A, miR-29a level was significantly reduced in the majority of cholangiocarcinoma tissues compared to cancer-adjacent tissues (P<0.01), whereas decreased expression of miR29b/c did not reach statistical significance (S1 Fig).

To further characterize the expression of miR–29 in cholangiocarcinoma cells, we performed qRT-PCR in two tumor cell lines:FRH–0201, CCLP–1,in comparison with the human intrahepatic bile duct epithelial cell line HIBEC. Similar to the result from the tissues, miR-29a was down-regulated in the two cholangiocarcinoma cell lines (Fig 1B), whereas no significant differences were detectable in miR29b/c levels. (S1 Fig).

These data suggest that dysregulation of miR-29a might contribute to the tumorigenesis.

### TGF-β1 dependent downregulation of miR-29a in cholangiocarcinoma

Overexpression of TGF-β1 are associated with tumor progression and metastasis in cholangiocarcinoma[6–7]. Recent reports also showed that miR-29a can be regulated by TGF-β1 during liver fibrogenesis[26], therefore we hypothesized whether TGF-β1 facilitates tumorigenesis by reducing miR-29a expression in cholangiocarcinoma.

**Fig 2:**
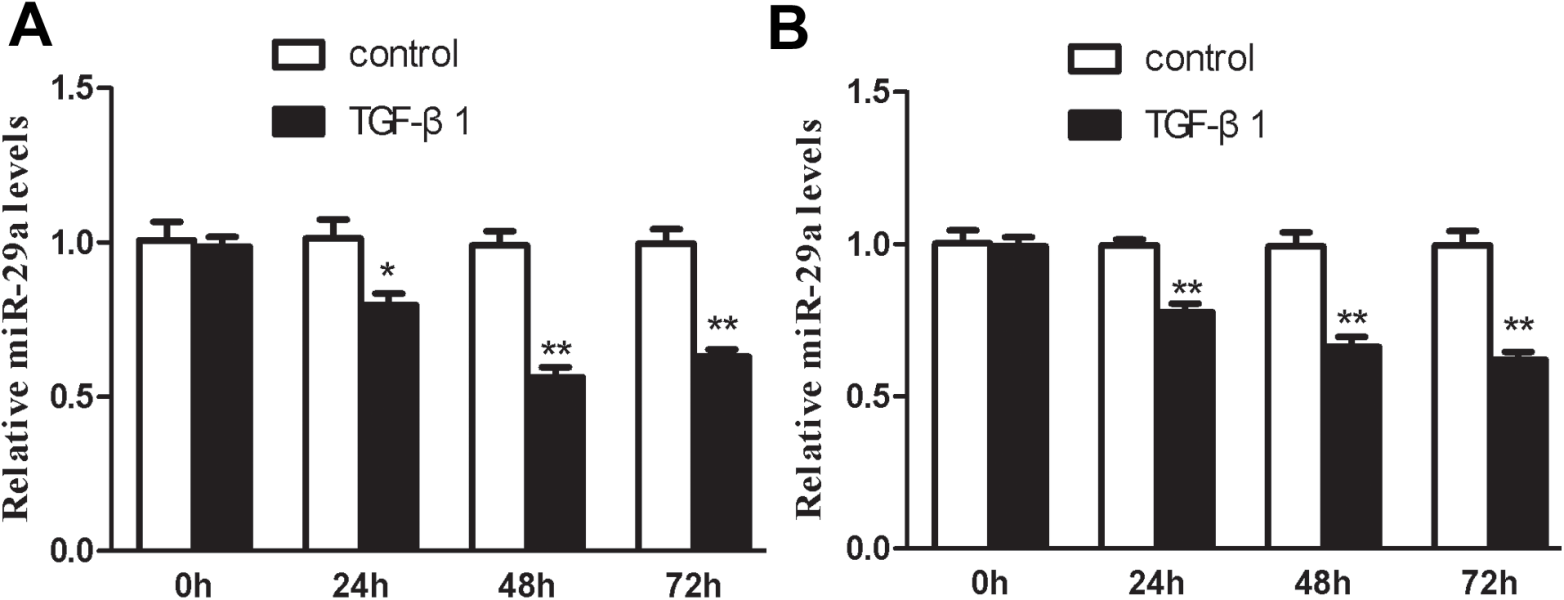
TGF-β1 reduced the expression of miR-29a in the two cholangiocarcinoma cell lines. (A and B) qRT-PCR was performed respectively in FRH–0201 and CCLP–1 cell lines after treatment with 5ng/ml TGF-β1 for 48h. Data are shown as mean±SD; *p<0.05;**P<0.01.

To verify this speculation, two independent cholangiocarcinoma cell lines(FRH–0201 and CCLP–1 cells)were stimulated with recombinant TGF-β1 (5ng/ml), and the expression of miR-29a was examined by qRT-PCR. As shown in Fig 2A and 2B, TGF-β1 dramatically reduced the expression of miR-29a in the two cell lines examined.

### Overexpression of miR-29a attenuates TGF-β1-mediated cholangiocarcinoma cell growth and metastasis

**Fig 3:**
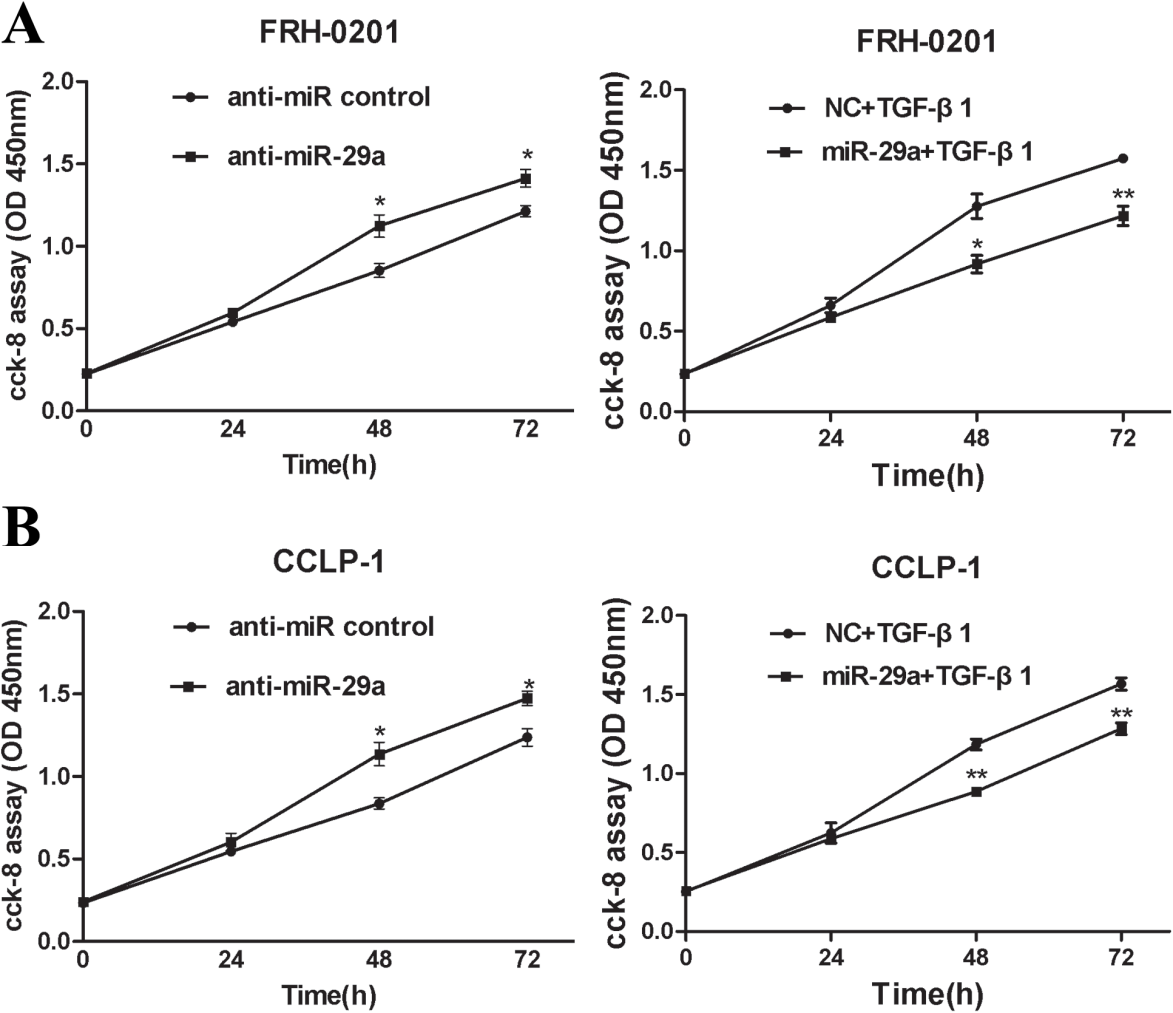
Up-regulation of miR-29a attenuates TGF-β1-mediated cholangiocarcin- oma cell growth. Cell proliferation assay was performed in FRH–0201 (A) and CCLP–1 (B) cells, 48h after transfection with anti-miR-29a. When the two tumor cell lines were treated with miR-29a mimic, cell proliferation assay was performed after additional treatment with 5ng/ml TGF-β1 for 48h. Data are shown as mean±SD; *p<0.05;**P<0.01.

The above findings prompted us to ascertain the biological effects of miR-29a in cholangiocarcinoma development. miR-29a level was highly increased or decreased respectively in FRH–0201 cells after transfection with miR-29a mimic or the miR-29a inhibitor (anti-miR-29a) (S2 Fig). This result was reproducible in CCLP–1 cells(S2 Fig). To investigate the role of miR-29a in cholangiocarcinoma cell growth, cell proliferation assay was performed in FRH–0201 and CCLP–1 cells. According to the data of the CCK–8 assay kit (Dojindo, Japan), we drew the absorbency curves at the wave length of 450nm. The cell proliferation curves showed that the growth rate of the two tumor cells were significantly increased in a time-dependent manner, compared to the negative control when transfected with inhibitors of miR-29a. Interestingly, overexpression of miR-29a counteracted TGF-β1-mediated cell proliferation (Fig 3A and 3B).

**Fig 4:**
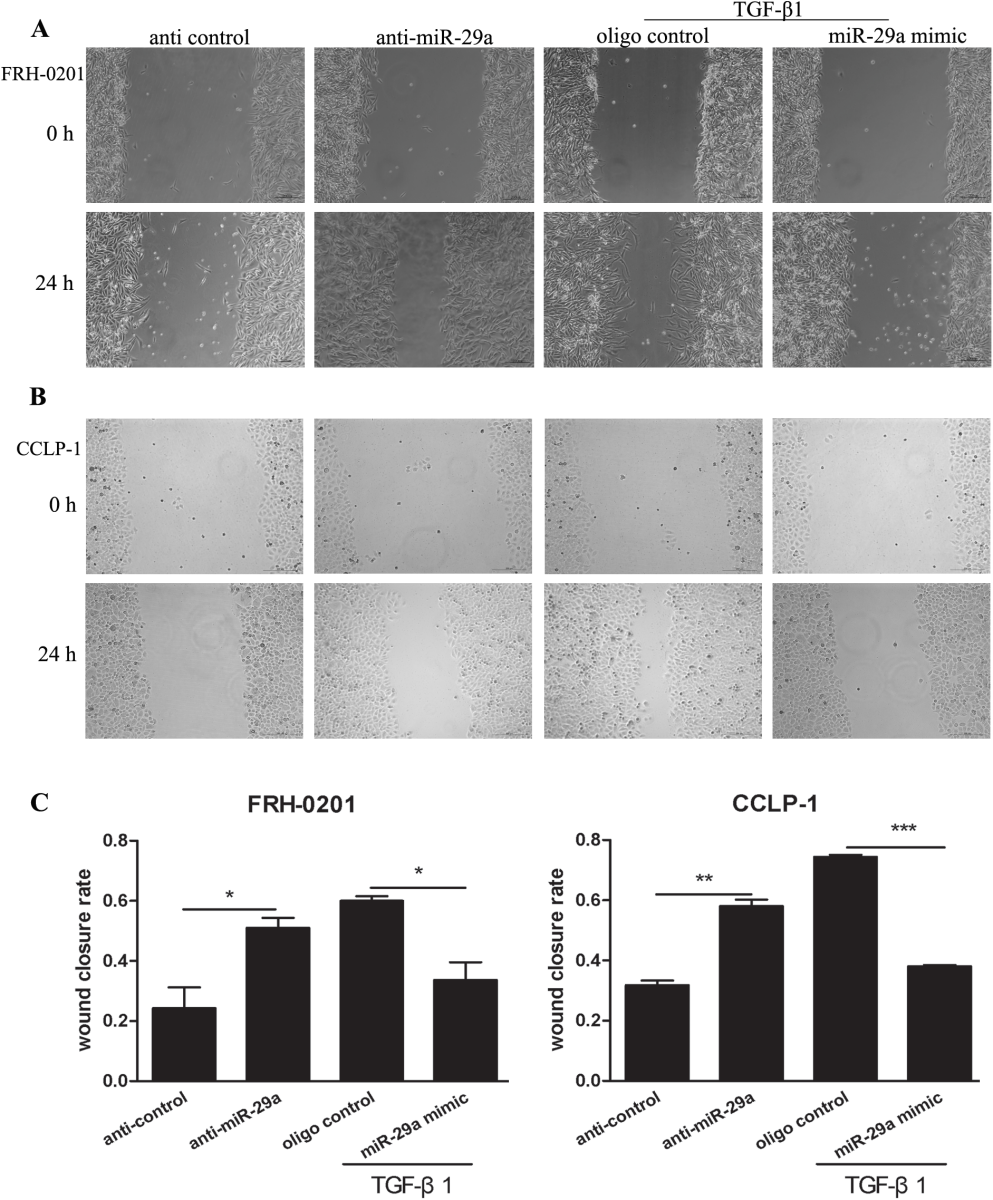
Enhanced expression of miR-29a attenuates TGF-β1-mediated cholangio- carcinoma cell metastasis. Wound healing assay was performed in FRH–0201 and CCLP–1 cells (A-C), 48h after transfection with anti-miR-29a. When the two tumor cell lines were treated with miR-29a mimic, wound healing assay was performed after additional treatment with 5ng ml^-1^TGF-β1 for 48h. Data are shown as mean±SD; *p<0.05;**P<0.01;*** P<0.001.

**Fig 5:**
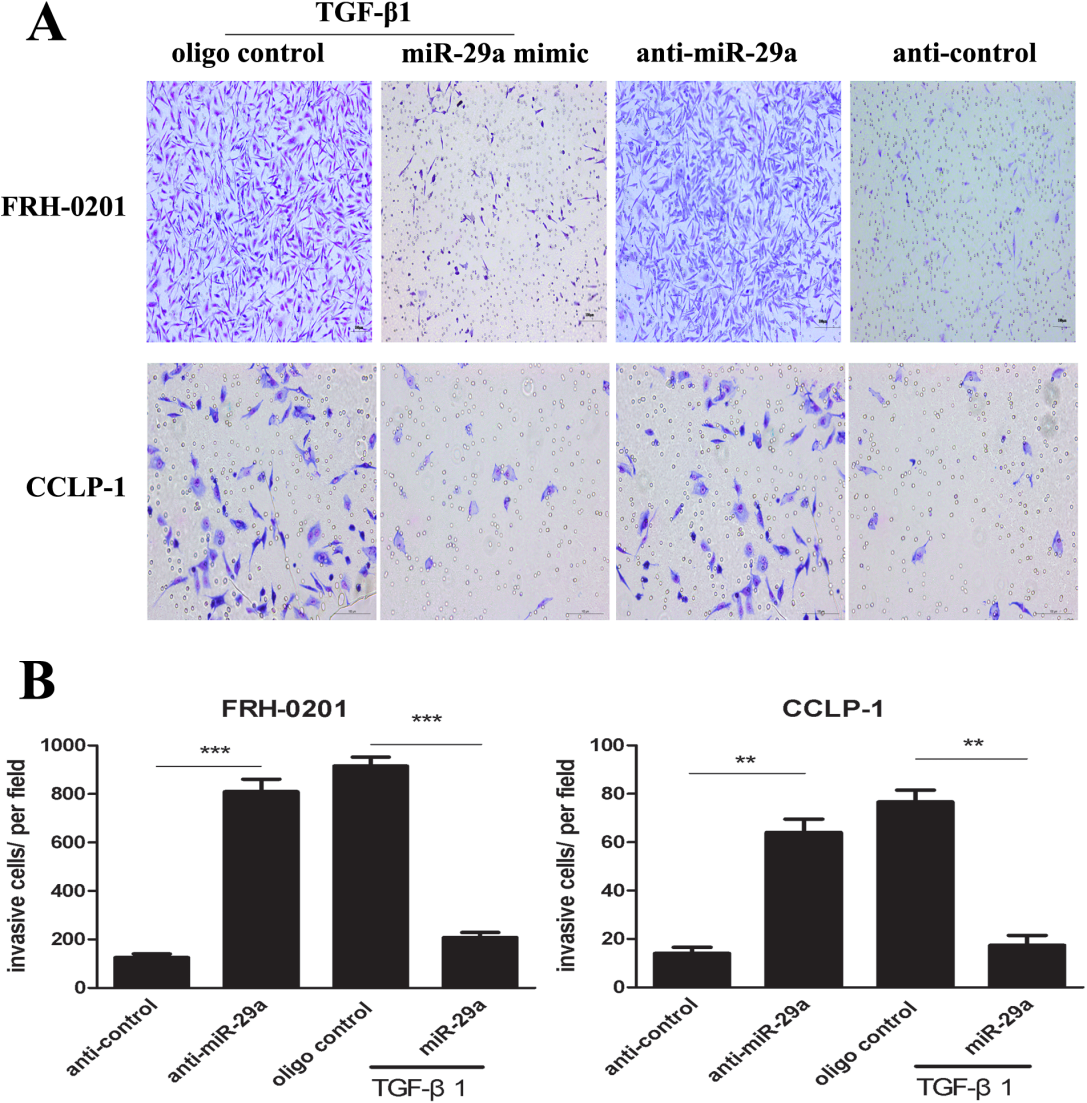
Transwell cell invasion assay. (A and B) Transwell assay was administrated in the two tumor cell lines, 48h after treatment with anti-miR-29a. When the two tumor cell lines were treated with miR-29a mimic, the migrating cells were counted after additional incubation with 5ng ml^-1^TGF-β1 for 48h. Data are shown as mean±SD; **P<0.01;*** P<0.001.

Moreover, wound healing assay and transwell assay were used to evaluate the effect of miR-29a overexpression on the migration and invasion mediated by TGF-β1 in tumor cells. As a result, miR-29a inhibitor increased the migration and invasion respectively in the two tumor cells (Figs 4 and 5). Importantly, up-regulation of miR-29a by treatment with miR-29a mimic attenuated TGF-β1-mediated cell migration and invasion. Collectively, these results indicate that miR-29a contributes to TGF-β1-induced cholangiocarcinoma progression.

### HDAC4 is a direct target of miR-29a

We then explored the underlying mechanism by which miR-29a functions in cholangiocarcinoma. Among the hundreds of miR-29a targets predicted by Targetscan, HDAC4 was selected for further study, because the seed sequence in HDAC4 mRNA 3′-UTR completely match to miR-29a, more importantly, it has been proved to be linked with tumorigenicity. HDAC4 is a confirmed target of miR-29b in mouse osteoblast differentiation[27], but it has not been validated in tumor cells.

**Fig 6:**
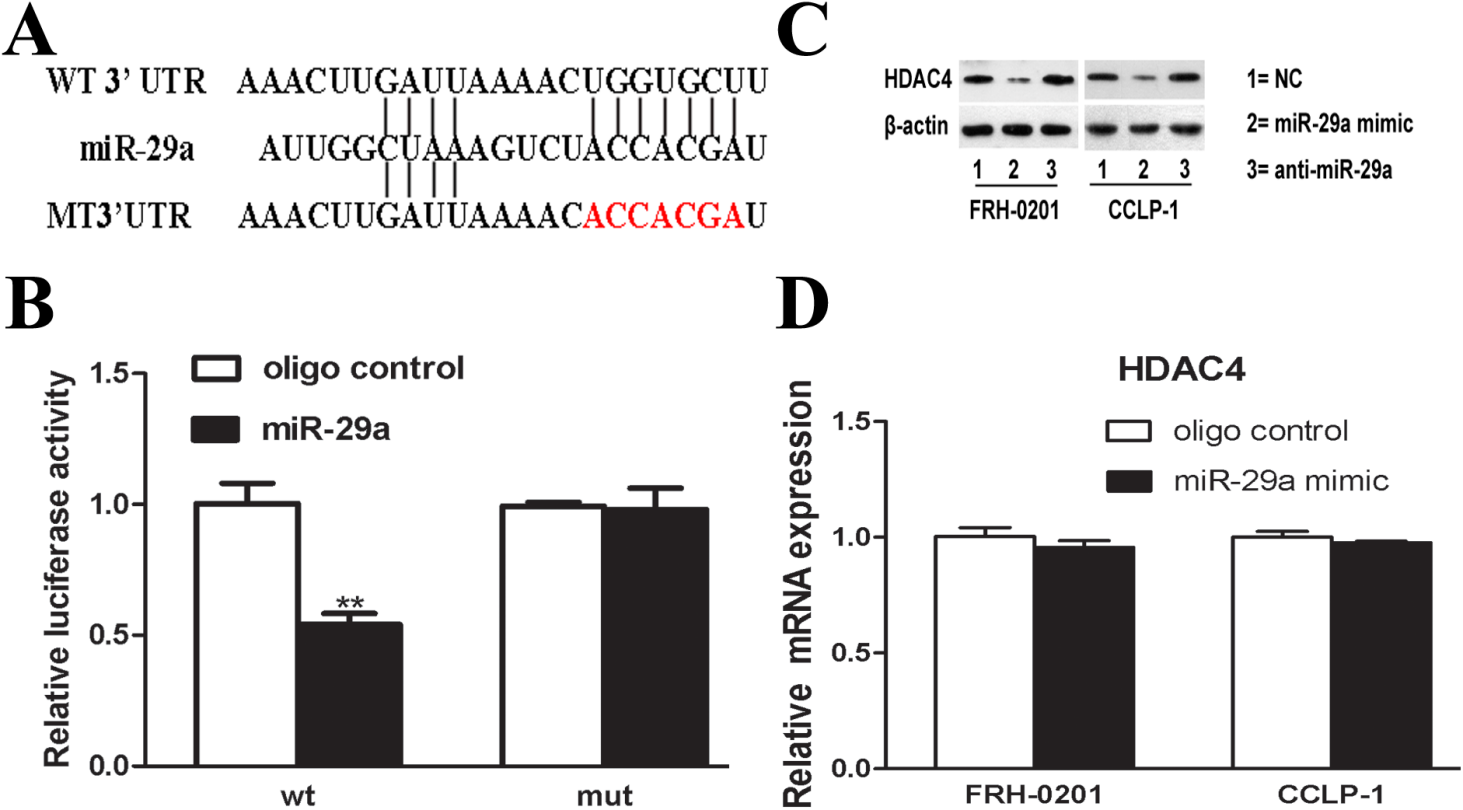
HDAC4 is a target of miR-29a. (A) Predicted putative (upper) and mutated (lower, shown in red) binding sequence in the 3’UTR of HDAC4 mRNA, displayed from 5’ to 3’. (B) The relative luciferase activity. miR-29a mimic (50nM) or NC (50nM) were co-transfected with wt or mut–3’UTR luciferase reporter. (C) Western blot analysis was performed in FRH–0201 and CCLP–1 cells. HDAC4 was reduced by miR-29a mimic (50nM), compared with NC. (D) The relative mRNA expression of HDAC4. Data are shown as mean±SD; **P<0.01.

To confirm that HDAC4 was indeed a direct target of miR-29a in human cells, we used luciferase reporter constructs containing wild-type and mutant HDAC4-3′-UTRs, both with the putative binding site of miR-29a (Fig 6A). As shown in Fig 6B, miR-29a decreased the luciferase activity of the reporter vector containing the wild-type HDAC4 3′-UTR compared to the control (P < 0.05), in contrast, only a minimal effect on the mut-HDAC4 3′-UTR reporter was evident.

In addition, further research showed that transfection with miR-29a mimic decreased HDAC4 protein in both two cell lines (Fig 6C), in contrast, miR–29 inhibitor resulted in the up-regulation of HDAC4 protein. Interestingly, no significant change in HDAC4 mRNA levels was evident(P > 0.05) (Fig 6D). These data suggest that miR-29a negatively regulated HDAC4 expression at the posttranscriptional level by directly targeting HDAC4 mRNA–3′UTR seed sequence.

**Fig 7:**
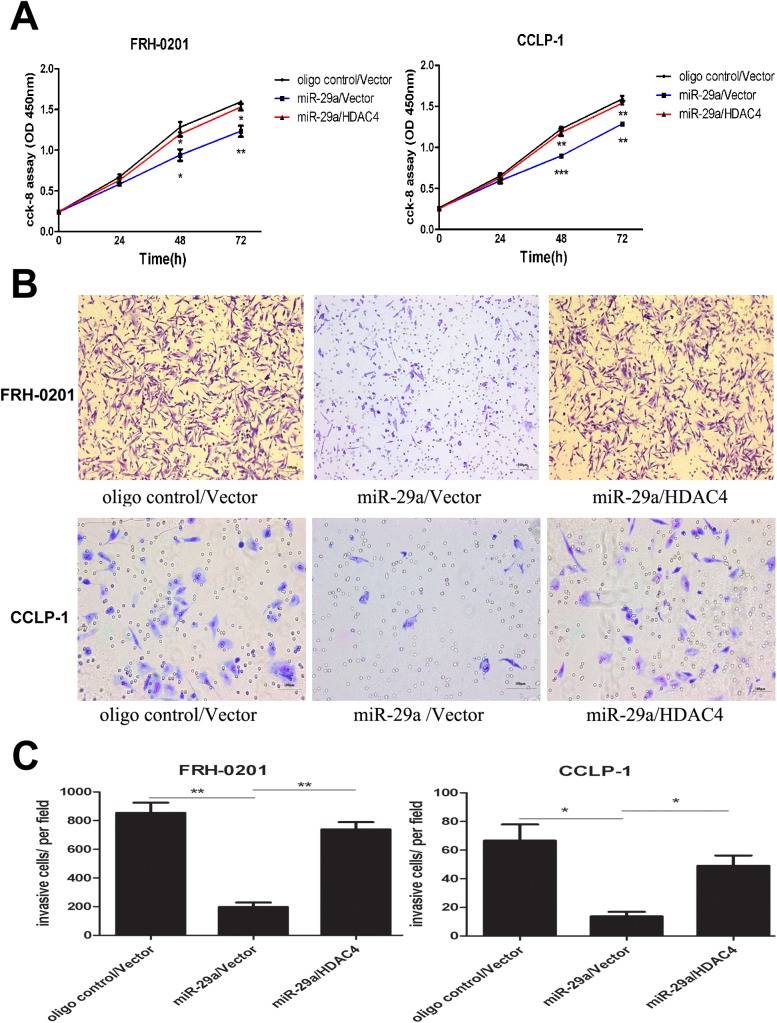
Enhanced HDAC4 expression abrogated the tumor suppressive function of miR-29a in cholangiocarcinoma. (A) Cell proliferation assay was performed in FRH–0201 and CCLP–1 cells, 48h after transfection with NC, miR-29a mimic or miR-29a mimic/pcDNA3.1- HDAC4. (B and C) Transwell assay was carried out in the two tumor cell lines, 48h after treatment with NC, miR-29a mimic or miR-29a mimic/pcDNA3.1- HDAC4. Data are shown as mean±SD; *p<0.05;**P<0.01;*** P<0.001.

We next determined the biological role of HDAC4 in cholangiocarcinoma cells. The CCK–8 assay data showed that restoration of HDAC4 counteracted miR-29a-mediated inhibition of cell proliferation (Fig 7A). Similarly, enhanced HDAC4 expression abrogated the anti-metastatic role of miR-29a (Fig 7B and 7C).

**Fig 8:**
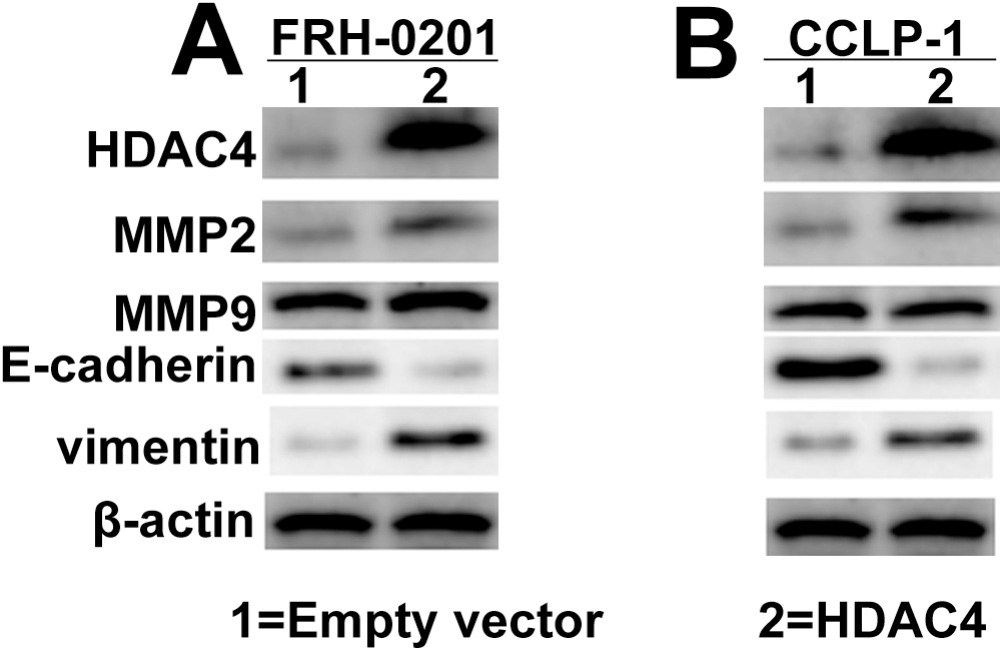
HDAC4 overexpression induces an EMT phenotype. (A) Enhanced HDAC4 expression led to increased MMP2 and Vimentin, decreased E-cadherin expression in FRH–0201 cells and CCLP-1cells (B).

Given that MMPs and EMT were involved in tumor cells metastasis, we further investigated the link between HDAC4 expression and E-cadherin, Vimentin, MMP2 and MMP9 expression. Western blot analysis showed that overexpression of HDAC4 increased MMP2 and Vimentin, decreased E-cadherin expression in FRH–0201 cells, however, there was no significant change in MMP9 level(Fig 8A). This result was reproducible in CCLP–1 cells (Fig 8B).

Taken together, these data suggest that TGF-β1-mediated miR-29a inhibition may contribute to cholangiocarcinoma cell proliferation and metastasis, which are partly associated with HDAC4-induced EMT.

## Discussion

Cholangiocarcinoma, associated with high mortality and a poor prognosis, is one of the most deadly cancers. Although remarkable improvement in cholangiocarcinoma therapy, local tumor growth, early metastasis and direct hepatic invasion remain to be major challenges in cancer treatment. However, the molecular mechanism that modulate the process of carcinogenesis remains largely unknown.

In the present study, downregulation of miR-29a was confirmed in clinical cholangiocarcinoma tissues compared with adjacent non-tumor tissues, as well as in the two tumor cell lines. Since aberrant miRNA expression contributes to tumor initiation and progression [15–17], thus the mechanisms inducing dysregulation of miR-29a expression in cholangiocarcinoma are of significant interest.

TGF-β1 has been revealed to play key roles in regulating mammary carcinoma initiation, progression and metastasis[3–7]. It has been confirmed that mutations in components of the TGF-β1 signaling pathway promote the malignant phenotype of a given tissue in pancreatic cancer and colon carcinoma[28,29]. For cholangiocarcinoma, TGF-β1 acts as a promoter of tumorigenesis through Sp1-dependent transcriptional activation of vascular endothelial growth factor(VEGF) or Snail activation[6,7]. In this report, we found expression of TGF-β1 notably increased in cholangiocarcinoma clinical specimens (S3 Fig), which is consistent with previous studies[6,7]. Furthermore, it could reduce miR-29a level in the both two tumor cell lines. In addition, upregulation of miR-29a suppresses tumor cells proliferation, migration and invasion, whereas silencing of miR-29a promotes TGF-β1-induced tumor progression. Although TGF-β1-dependent downregulation of miR-29a was demonstrated to be associated with oncogenesis, the underlying molecular mechanism for the regulation is currently unknown. miRNAs expression are always regulated by binding to the promoter or regulated process of microRNA precursors[30–33]. Further investigations are needed to elucidate whether TGF-β1 direct effects on the promoter regions or in cooperation with other transcription factors in regulating miR-29a expression.

As reported previously, miR-29a plays complex roles in various models of tumorigenesis [22–24]. MiR-29a acts as a tumor promoter in chronic lymphocytic leukemia (B-CLL) and acute myeloid leukemia (AML) [34,35], whereas in lung, pancreatic, and gastric cancer it functions as a tumor suppressor [22,23]. Thus, the roles played by miR-29a differ according to the cellular background. In our research, miR-29a acted as a tumor suppressor in cholangiocarcinoma. Previous reports have showed that MMP–2, Bcl–2 and Mcl–1 are direct targets of miR-29a in regulating tumor progression in different cancer cells[24,36,37]. Among the hundreds of predicted targets, HDAC4 was confirmed as a direct target of miR-29a, by various methods including bioinformatics, dual-luciferase activity report system and functional studies. Our data revealed that HDAC4 expression was negatively regulated by miR-29a at the posttranscriptional level.

Histone deacetylases (HDACS) regulate the expression levels of many proteins involved in both initiation and progression of cancer[38,39]. Aberrant HDAC expression is associated with carcinogenesis[40,41]. HDAC inhibitors (HDACIs) have been reported to induce apoptosis or trigger cell cycle arrest of cholangiocarcinoma cells[42,43]. HDAC4 belongs to class II of the HDACs[44]. A later study showed that HDAC4 became associated with Sp1 at the proximal p21 promoter, and promoted cancer cell growth via repression of p21 in an Sp1-dependent manner[38]. Yuan et al found that downregulation of miR-200a enhanced the proliferation and migration of hepatocellular carcinoma cells, by targeting HDAC4[25]. In our report, knockdown of HDAC4 suppressed cell proliferation and metastasis in vitro, which phenocopied the consequence of enhanced miR-29a expression, in contrast, reintroduction of HDAC4 partially mitigated miR-29a-mediated inhibition of cell proliferation and metastasis. Our observation suggest that the aberrant TGF-β1 expression in cholangiocarcinoma may led to reduced miR-29a level, which in turn affects the histone acetylation level and thereby facilitates carcinogenesis and tumor progression. However, the responsible mechanisms involved remain unknown. EMT is associated with the invasion and metastasis in different tumor cells[45]. In the present study, we found that enhanced HDAC4 expression led to dysregulation of hallmarks of EMT.

It is suggested that, the increased level of TGF-β1 in cholangiocarcinoma is responsible for the inhibition of miR-29a,followed by the activation of HDAC4 signaling, which may in turn promote EMT. During these complex processes, MMP–2 might also play a crucial role. Our data provide novel insight into the mechanism of TGF-β1/miR-29a/HDAC4 pathway in the pathogenesis of cholangiocarcinoma and provide new therapeutic targets for cholangiocarcinoma.

## Supporting Information

S1 Fig The expression of miR-29b and miR-29c in cholangiocarcinoma samples and cell lines. (A and B) The average expression level of miR-29b /miR29c was measured in forty human cholangiocarcinoma tissues and matched cancer-adjacent (normal) tissues. (C and D) Expression of miR-29b/miR29c in the human intrahepatic bile duct epithelial cell line HIBEC and two cholangiocarcinoma cell lines. Data are shown as mean±SD.
(TIF)

S2 Fig The relative expression levels of miR-29a. (A and B) The expression levels of miR-29a after transfection with miR-29a mimic(50 nM) and anti-miR-29a (100 nM) respectively in FRH–0201 cells. (C and D) The levels of miR-29a were measured in CCLP–1 cells.
(TIF)

S3 Fig The expression of TGF-β1 is increased in cholangiocarcinoma tumors (T) compared with the tumor-adjacent tissues (N) by Western blotting. (TIF)
