# Supplementary figures and images for: TGF-β1 Reduces miR-29a Expression to Promote Tumorigenicity and Metastasis of Cholangiocarcinoma by Targeting HDAC4
Source: PLoS One. 2015 Oct 6;10(10):e0136703. doi: 10.1371/journal.pone.0136703 (PMC4595145; doi:10.1371/journal.pone.0136703)

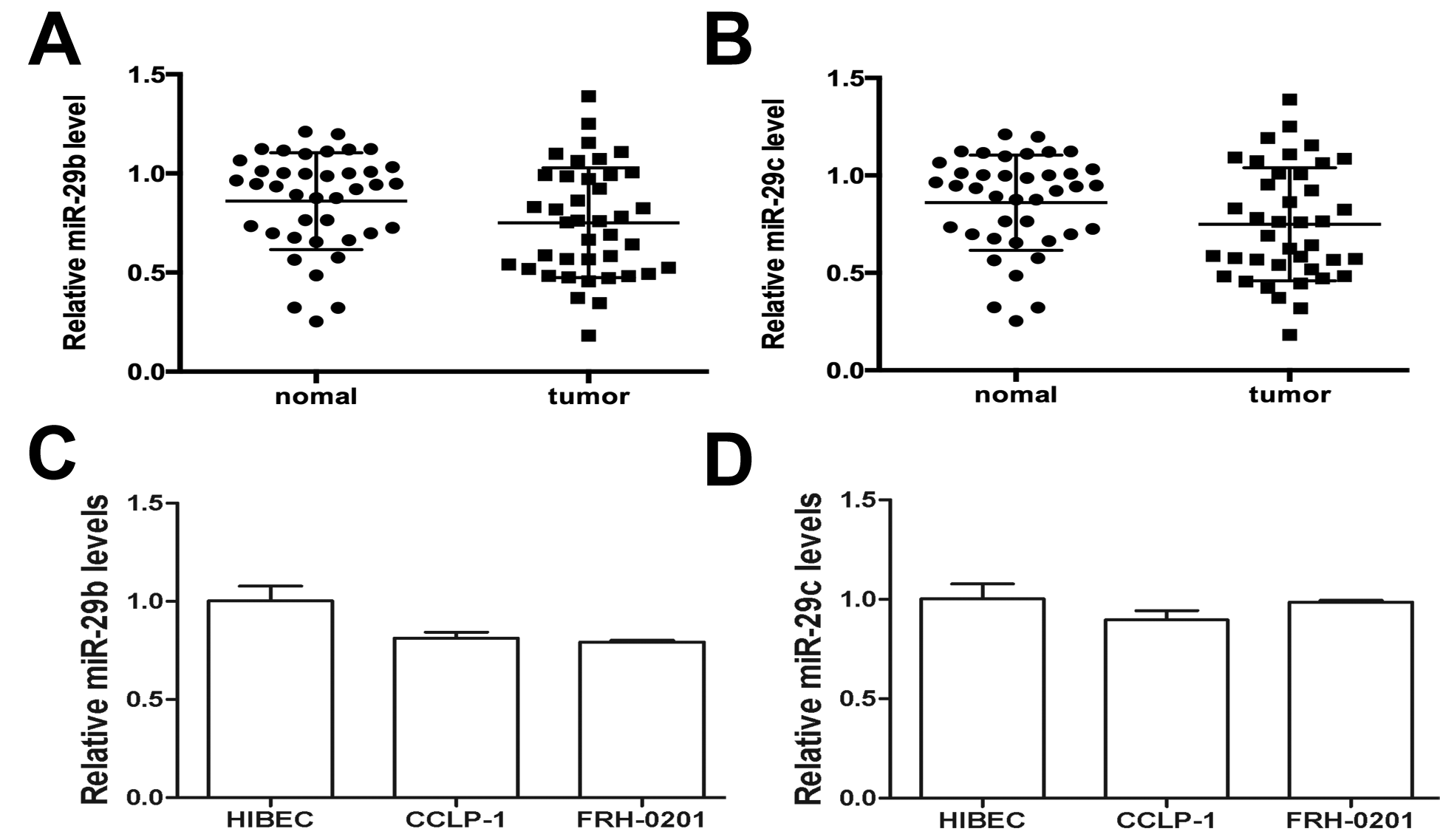

Supplement: S1 Fig — (A and B) The average expression level of miR-29b /miR29c was measured in forty human cholangiocarcinoma tissues and matched cancer-adjacent (normal) tissues. (C and D) Expression of miR-29b/miR29c in the human intrahepatic bile duct epithelial cell line HIBEC and two cholangiocarcinoma cell lines. Data are shown as mean±SD. (TIF) [file pone.0136703.s001.tif]

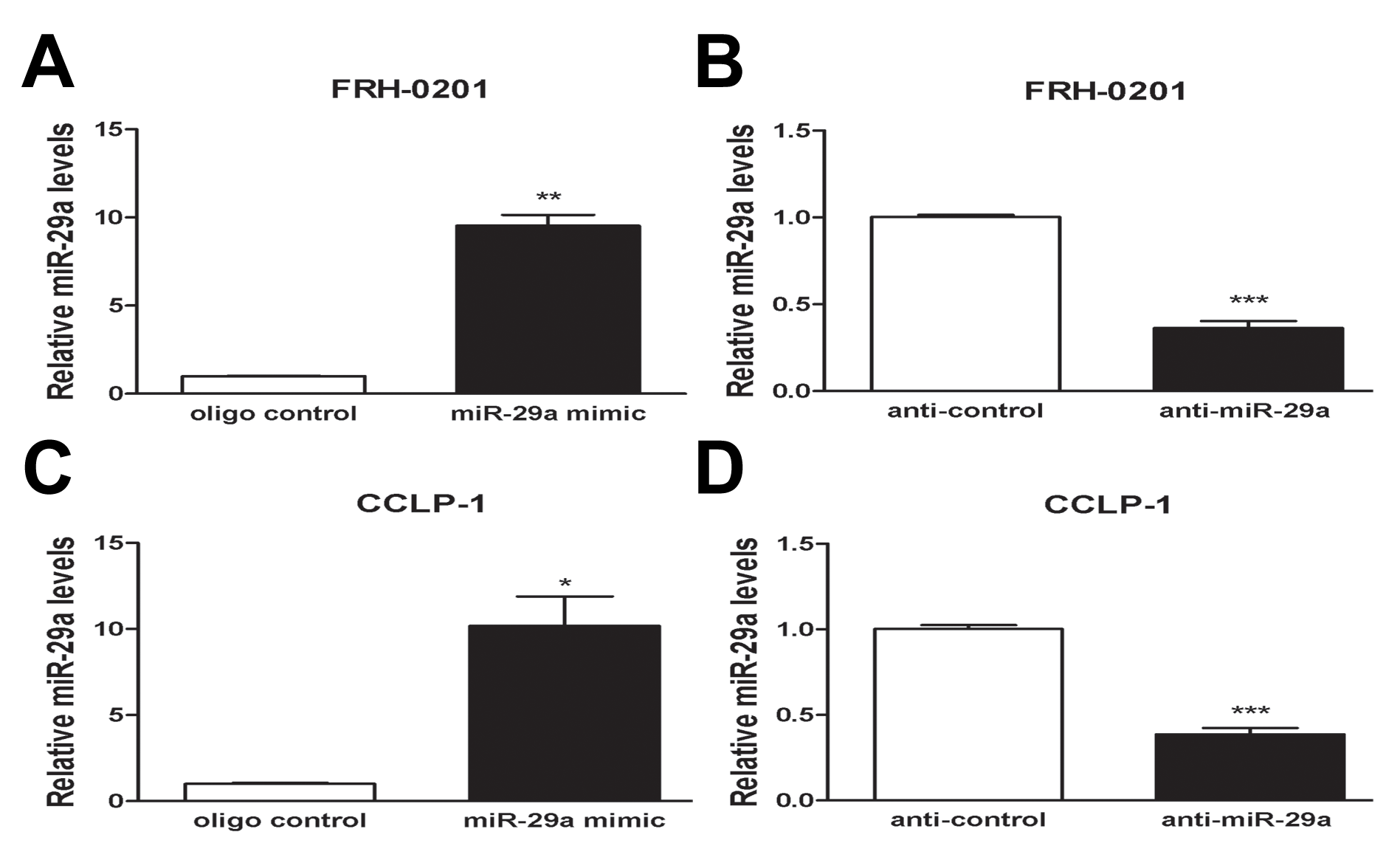

Supplement: S2 Fig — (A and B) The expression levels of miR-29a after transfection with miR-29a mimic(50 nM) and anti-miR-29a (100 nM) respectively in FRH–0201 cells. (C and D) The levels of miR-29a were measured in CCLP–1 cells. (TIF) [file pone.0136703.s002.tif]

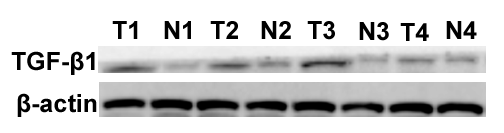

Supplement: S3 Fig — (TIF) [file pone.0136703.s003.tif]
